# Field Propagation Experiments of Male African Savanna Elephant Rumbles: A Focus on the Transmission of Formant Frequencies

**DOI:** 10.3390/ani8100167

**Published:** 2018-09-30

**Authors:** Anton Baotic, Maxime Garcia, Markus Boeckle, Angela Stoeger

**Affiliations:** 1 Mammal Communication Lab, Department of Cognitive Biology, University of Vienna, Vienna 1090, Austria; maxime.garcia@ymail.com; 2 ENES Lab, Neuro-PSI, CNRS UMR 9197, University of Lyon/Saint Etienne, 42023 Saint Etienne, France; 3 Department of Psychology, University of Cambridge, Cambridge CB2 3EB, UK; markus.boeckle@gmail.com; 4 Department of Psychotherapy, Bertha von Suttner University, St. Poelten 3100, Austria

**Keywords:** African savanna elephant, rumble, vocalization, formant, propagation

## Abstract

**Simple Summary:**

African savanna elephants are highly social and exhibit a complex vocal communication system. They use a low-frequency contact call (termed ‘rumble’) to maintain social contact over long distances. As sound travels through the environment, however, its intensity level decreases. We used specialized computer software to manipulate acoustic components in male rumbles and simulated different body sizes (large and small). The rumbles were broadcasted and re-recorded at different distances at the Addo Elephant National Park, South Africa. This propagation experiment enabled us to investigate which acoustic components and information content can be transmitted efficiently up to 1.5 km. The results confirm that male rumbles potentially encode information about body size, yet transmission success decreased with distance. Our findings inform on how the environment can influence propagation of savanna elephant rumbles and what kind of information might be transmitted successfully over distance.

**Abstract:**

African savanna elephants live in dynamic fission–fusion societies and exhibit a sophisticated vocal communication system. Their most frequent call-type is the ‘rumble’, with a fundamental frequency (which refers to the lowest vocal fold vibration rate when producing a vocalization) near or in the infrasonic range. Rumbles are used in a wide variety of behavioral contexts, for short- and long-distance communication, and convey contextual and physical information. For example, maturity (age and size) is encoded in male rumbles by formant frequencies (the resonance frequencies of the vocal tract), having the most informative power. As sound propagates, however, its spectral and temporal structures degrade progressively. Our study used manipulated and resynthesized male social rumbles to simulate large and small individuals (based on different formant values) to quantify whether this phenotypic information efficiently transmits over long distances. To examine transmission efficiency and the potential influences of ecological factors, we broadcasted and re-recorded rumbles at distances of up to 1.5 km in two different habitats at the Addo Elephant National Park, South Africa. Our results show that rumbles were affected by spectral–temporal degradation over distance. Interestingly and unlike previous findings, the transmission of formants was better than that of the fundamental frequency. Our findings demonstrate the importance of formant frequencies for the efficiency of rumble propagation and the transmission of information content in a savanna elephant’s natural habitat.

## 1. Introduction

Many group-living mammal species have developed complex social and spatiotemporal association patterns [1]. Depending on the costs involved to maintain social cohesion, groups may temporarily split and vary in size as they move through the environment, helping balance the costs and benefits of grouping. Spatial coordination in such dynamic fission–fusion societies requires communication and information transfer/exchange between multiple signalers and receivers in their active space [2,3]. Vocal signals may transmit information about a caller’s identity and physical attributes (age, body size, and sex) and are particularly important for facilitating social recognition and mating success [4]. In this context, a key objective will often be to maximize the propagation distance in the animal’s natural habitat [5], which in turn depends on the receiver’s ability of assessing a vocal signal’s degradation level to determine a nearby caller’s distance (termed ‘ranging’) and to mediate interindividual spacing [6].

In general, as sound waves propagate through the environment, the spectral and temporal structure degrades progressively with distance, yielding a 6 dB attenuation of the signal amplitude (source intensity that corresponds to sound pressure) per distance doubling (termed ‘spherical spreading’) under frictionless open field conditions, i.e., far from any obstructions. This potentially constrains the signal’s active space and thus affects decoding acoustic information by receivers [7,8,9]. Further factors causing ‘excess attenuation’ are ambient noise, fluctuations or changes of atmospheric conditions, vegetation strata, topography, and reverberation (frequency-dependent repeated reflections of a signal). Attenuation of higher frequencies and reverberation are more pronounced in dense habitats due to tree trunks, branches, and foliage, whereas open field habitats possess fewer reflecting barriers. In open habitats, however, irregular atmospheric conditions, such as high wind speeds or temperature, may affect sound propagation [7,8,9,10,11,12]. Studies in baboons [13] and birds [14,15] demonstrated the beneficial effect of sound reflection on sound transmission. Accordingly, reflected sound waves can contribute energy to the source sound wave when both waves overlap and hence affect sound propagation. Ground effects are caused by constructive or destructive interference effects, between sound traveling from a source to a receiver and sound being reflected from the ground that occur at a receiver’s location resulting, respectively, either in enhancement or attenuation of a received sound pressure level SPL (usually given in decibels, dB) [16]. But signal amplitude/SPL alone has been suggested not to be a reliable acoustic cue to assess the distance of a sound source as (1) a signaler could vary its amplitude when facing away from or towards a listener and (2) acoustic signals can be affected by the attenuation factors mentioned above [17,18,19]. These attenuation factors differ between habitats and influence the acoustic characteristics of signals due to overall temporal degradation, frequency-dependent attenuation, and degradation processes [7,8,12]. Additional temporal and spectral structures of a signal are more likely to contribute to a more complete set of acoustic cues for distance assessment and information decoding than only amplitude/SPL, as it has been demonstrated in frogs [20], birds [18], and mammals (e.g., elephant seals [21] and bison [22]). Overall, lower-frequency sound experiences less attenuation than higher-frequency sound [8,12,23,24]; various mammal species use low-frequency vocalizations to maintain social relationships with conspecifics over distance [25,26,27].

Low-frequency communication is well developed in the African savanna elephant (*Loxodonta africana*) [28], a socially and spatially flexible species [29,30]. Though they produce a range of different vocalizations, the low-frequency ‘rumble’ is the most frequently produced call type. The rumble is a harmonically-rich vocalization with frequency components near or in the infrasonic range, used for both short- and long-distance communication [28,31,32,33,34]. In savanna elephants, rumbles are known to transfer information about identity, sex, age, size, arousal, and reproductive state [32,34,35,36,37,38], and enable communication over long distances to coordinate movements and to maintain contact between spatially separated individuals [34,39].

In general, sound production mechanisms in mammal species can be explained by the ‘source–filter’ theory, which states that a vocal signal is generated by vibrations of the vocal folds in the larynx (source) and modified acoustically by the vocal tract (filter) between the larynx and the mouth [40]. The dimensions of the vocal folds (length and thickness) and their average vibration rate define the fundamental frequency (*f_o_*) [40,41]. Comparative perceptual studies in other mammals, such as in deer [42], koalas [43], and domestic dogs [44], showed that vocal tract resonance frequencies (formants) are a reliable cue to body size and are therefore biologically relevant [4]. Vocal tract (VT) length and body size are anatomically correlated in savanna elephants [37,38]; VT length generally determines formant dispersion (FΔ, average spacing between successive formants). This in turn provides acoustic information about body weight and mass. The longer the vocal tract, the lower the formants and the narrower the overall FΔ [45].

The influence of an animal’s body size on its sound production and vocal performance is an important biological constraint [46]. In a wide range of terrestrial mammals with pronounced sexual size dimorphism, it has been suggested that individuals, typically males, are able to produce vocalizations with lowered formants and formant dispersions. This may be achieved, for example, by extending the VT (e.g., red deer *Cervus elaphus*), by utilizing additional resonators, or by developing nasal proboscises (e.g., elephant seal *Mirounga* sp.). These adaptations help broadcast an exaggerated impression of body size in vocalizations involved in reproductive contexts (for a comprehensive review see the literature [47]). The savanna elephant’s nasal vocal tract is exceptionally elongated compared to its oral vocal tract and hence occupies a special position amongst mammal vocal production. Savanna elephants follow the basic mechanism of mammalian sound production [48] and can make use of their nasal vocal path to emit rumbles. This enables them to lower their formant frequencies compared to orally emitted rumbles, making nasal rumbles particularly suitable to communicate over long distances [49]. Nonetheless, the adaptive significance of formant modulations in savanna elephants remains unknown: do the very low formant frequencies of rumbles reflect sexual (or other social) selection pressures to sound larger, or do they reflect natural selection pressures to maximize call propagation distances? Note that both selection pressures could be operating and are not necessarily mutually exclusive.

In the present study, we applied a resynthesis technique on male savanna elephant social rumbles to create playback stimuli with different formant variations for field propagation experiments. We manipulated the most consistent formant locations (i.e., the first and second formants, *F1* and *F2*, respectively) while leaving the *f_o_* unchanged, and generated stimuli with apparent vocal tract length differences simulating different male body sizes (i.e., two different maturity groups). This approach was designed to determine whether lower formants propagate further than higher formants in comparable conditions while other parameters remain unchanged, and to evaluate the active communication range (information transmission) during daytime conditions. By broadcasting and re-recording rumbles at increasing distance, we quantified the extent to which size-related information degrades with increasing distance in a savanna elephant’s natural environment. Our experimental and analytical approach also enabled us to assess the impact of two different habitats and environmental conditions on transmission properties in this long-distance call.

## 2. Materials and Methods

### 2.1. Sound Recordings of Playback Stimuli

The rumbles used in the propagation experiments originated from four adult male savanna elephants from four South African private elephant reserves (Table 1) recorded in social (nonreproductive) contexts (e.g., when individuals maintained vocal contact with other group members during free-roaming and browsing activities in areas of 3 to 45 km^2^). Acoustic recordings were conducted throughout the day between 7 AM and 5 PM by following the animals on foot accompanied by the keepers, without any interaction with the animals. Recording distances of the selected vocalizations were less than 10 m from the calling individual.

We used an omni-directional Neumann KM183 condenser microphone (fitted with a windshield), modified for recording frequencies below 20 Hz (flat-recording down to 5 Hz). The microphone was connected to a Sound Devices 722 (frequency response: 10 Hz–40 kHz, +0.1, −0.5 dB (gain controls centered); Sound Devices LLC, Reedsburg, WI, USA), recording rumbles with a 48 kHz sampling rate and an amplitude resolution of 16 bits.

### 2.2. Study Site and Conditions

Transmission experiments were conducted at the Addo Elephant National Park (AENP; 33°30’ S, 25°45’ E), Eastern Cape Province of South Africa, covering an area of approximately 270 km^2^ (Roxanne Erusan, AENP Scientific Services, personal communication). The AENP is located in the endemic-rich, xeric succulent thicket vegetation (e.g., succulents, deciduous shrubs, lianas, and grass) of the Eastern Cape [50]. AENP hosts a variety of habitats from areas with a high density of thorny thicket vegetation (the most prominent plant is the succulent ‘Spekboom’, *Portulacaria afra*) up to 3 m high (rarely higher) to extensive areas of open grasslands [51]. AENP consists of a series of undulating hills with an altitude range of 60 to 350 m above sea level [52].

Propagation trials were carried out at two locations: the ‘Rooidam’ section, representing a densely vegetated habitat with slight height differences ranging from 1 to 13 m, and at the ‘Gorah’ section, an open grassland habitat with greater height differences (0–59 m). A total of six recording days (three per habitat) were conducted on 26, 30, and 31 March 2017, and on 1, 2, and 4 April 2017, respectively. Sound propagation in the African savannah is best after sunset and 1–2 h before sunrise when ground level temperature cools down [53]. Experiments started at 05:30 a.m. at the earliest (due to security reasons), and finished at the latest at about 09:00 a.m. under dry and low wind conditions.

### 2.3. Experimental Design

#### 2.3.1. Preparation of Playback Stimuli

The focus of this study was on long-range nasal rumbles. The predicted formant locations for nasal rumbles are *F1* = 35.0 Hz and *F2* = 105.0 Hz using an estimated vocal tract (VT) length of 2.5 m [32]. In general, in savanna elephant vocalizations only the first two formants are consistently present [54,55,56]. After visual spectrographic inspection of our sound recordings in Praat [57], we preselected one nasal rumble per individual with low levels of background noise, clear *f_o_* and upper harmonics including *F1* and *F2*. Rumbles with formants equal or below the predicted values mentioned above were classified as nasally emitted.

For each stimulus a stop Hann band (0–5 Hz) and pass Hann band (6–200 Hz) filter was applied in Praat. This enabled proceeding solely with the relevant frequency ranges and removed as much background noise as possible.

We used the ‘To Formant (keep all)’ Praat function to inspect each rumble visually and automatically track formants. This yielded the optimal analysis settings (e.g., formant number: 2, maximum formant: 110 Hz, window length: 0.3, pre-emphasis: 5 Hz; note that these setting differed according to the rumble used).

Formants were modified using a custom-written script applying the source–filter resynthesis technique in Praat. Each rumble’s formant locations were down- and upshifted by 25%. We additionally used a 0% shift condition, where formants remained in their original position to control for the resynthesis procedure (i.e., original and ‘0% shifted’ rumbles are identical). Other acoustic parameters (e.g., *f_o_*, duration) remained unchanged for all three variants. All sound stimuli were normalized to a peak intensity of 0.99, yielding a test set of 12 WAV sound files, i.e., three different shift conditions per individual (see Table S1 for the measured formant values).

#### 2.3.2. Field Recordings

To examine the transmission success of rumbles with increasing distance, we played back and re-recorded 504 male rumbles in total (252 per habitat) at 50 m, 100 m, 200 m, 400 m, 800 m, 1000 m, and 1500 m. We used a custom-made subwoofer INFRA10 (dimension: 198 × 166 × 171 mm, weight: ~300 kg) linked to a rechargeable multipower MP45-12 lead-acid battery and a JL Audio HD1200/1 audio amplifier, connected to a 722 Sound Device HDD recorder to broadcast the playback stimuli. The INFRA10 is constructed for low frequencies, giving a flat response from 10 to 200 Hz at peak sound pressure levels measured at 1 m from the source of 110 dB at 10 Hz (referenced to 20 μPa). Stimuli were played back in sequences (with one sequence consisting of all three shift variants) at 105 dB ± 1 dB at 1 m, measured with a NTi NG AL1 sound pressure level meter equipped with a calibrated NTi MiniSPL microphone (settings: max SPL with SPL/RTA mode and FLAT response (unweighted)). In addition, we compared the playback recordings with the original stimuli in order ensure that signals were broadcasted correctly. Re-recordings were conducted using the identical recording equipment and settings used for recording the stimuli. The recording level remained unchanged and the same for all recording distances during the experiments (set at 60.5 dB at the 722 Sound Device recorder).

**Figure 1:**
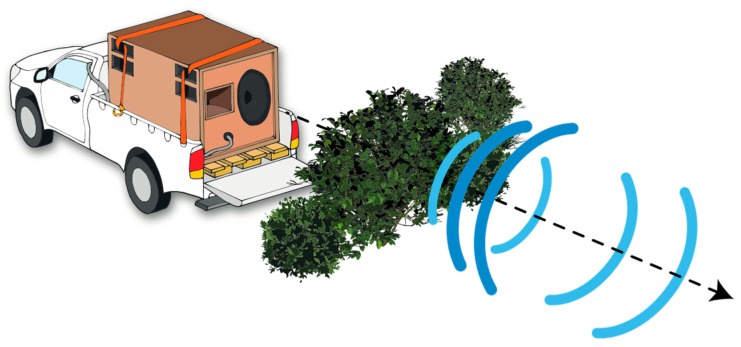
Schematic representation of the positioned infrasonic loudspeaker system at a densely vegetated location. The battery-supplied INFRA10 is mounted on the loading zone of a pick-up and operated inside the vehicle (broadcasting direction of playback stimulus indicated by dashed arrow).

**Figure 2:**
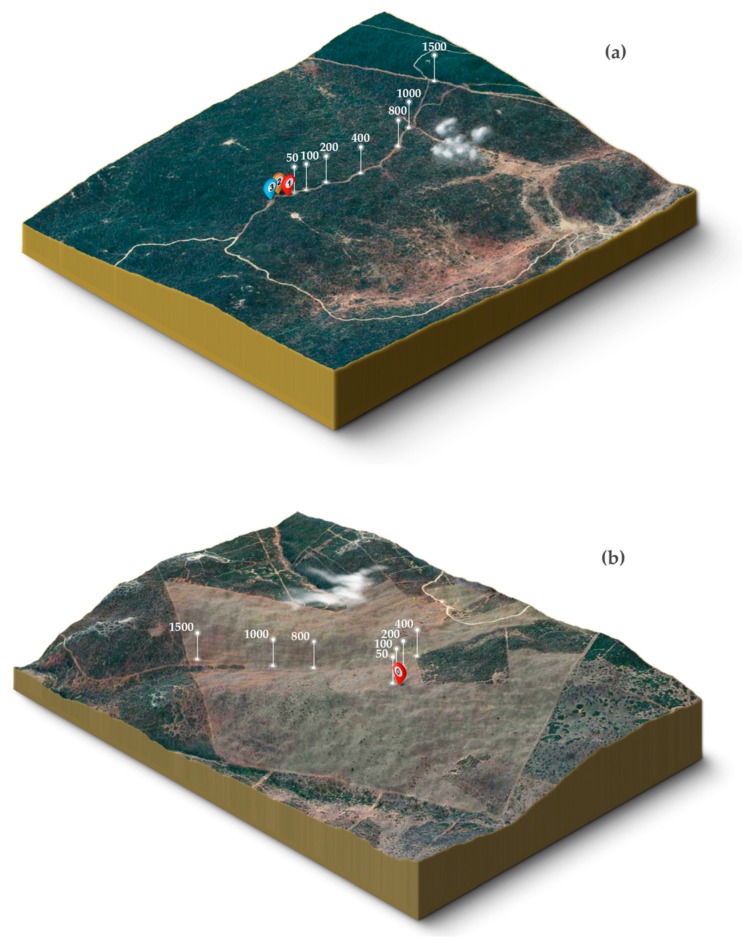
Topographical maps of both study sites, the densely vegetated (**a**) and the open (**b**) habitat. Vertical lines indicate the positions for each recording distance, ranging from 50 to 1500 m. The colored and numbered pins represent the fixed position of the subwoofer. In (**a**), pin #3 (in blue) corresponds to the position of the subwoofer for 1500 m, pin #2 (in orange) to the position of the subwoofer for 1000 m, and pin #1 for all other recording distances. Pin #0 (red) (**b**) represents the subwoofer’s position used to broadcast playback stimuli for all recording distances. Figures were generated using 3D Map Generator Terrain v1.4.2 (The Orange Box, Berlin, Germany) and Adobe Photoshop CC 2014 (Adobe Systems Incorporated, San Jose, CA, USA).

Two operators, using one Toyota Hilux pick-up each, conducted the field experiments. The subwoofer was mounted and transported on the loading zone of one pick-up, 1 m above the ground (see schematic representation in Figure 1). For each recording distance the subwoofer remained at a fixed position, while the second vehicle (the ‘recording vehicle’), from which the playback stimuli were recorded, was moved to the recording distances. During each trial, the playback and recording vehicles were in contact using a Stabo Freecomm 650 PMR radio set and mobile phones. To evaluate each recording distance’s position, we used the vehicles’ mileage counter and a Nikon Aculon ALL11 laser rangefinder. We additionally used the iOS application GPS Tour 2.0 [58] to track latitude, longitude, and altitude in order to verify the exact position and sea level for each distance (Figure 2). Landmarks were used to permanently mark each relevant distance for the entire experiment. Since GPS receivers use smoothed models of altitude to calculate elevation, we first converted these ellipsoidal heights into topographic heights (using a geoid height calculator [59]) before calculating absolute height differences between recording and subwoofer locations for each transmission distance (Table S2). The microphone was mounted and stabilized on a tripod outside the vehicle. To ensure direct orientation of the microphone towards the subwoofer’s broadcast direction, we used Bushnell powerview mid 10 × 42 binoculars for adjustments. Due to limited vision between both vehicles in the densely vegetated habitat (~3 m height of succulents), we used additional landmarks at each recording distance to point towards the subwoofer’s direction. To do this, the person operating the subwoofer positioned him- or herself centrally on top of the speaker (to be at the same level as the bush thicket), facing the subwoofer’s membrane and broadcasting direction. After visually confirming the subwoofer’s operator using a binocular, the person operating the recording equipment set the landmarks indicating the direction of the microphone towards the subwoofer for all follow-up trials.

For each distance, one playback sequence (consisting of the three formant shift variants 25% Down, 0% Unchanged, and 25% Up) for each individual (N = 4) was broadcasted, yielding a total of 84 playbacks across all seven recording distances per habitat and day. Depending on the occurrence or intensity of ambient noise (e.g., passing aircrafts), the respective playback sequence was repeated. To document atmospheric state per sequence, we used an anemometer PCE-THA 10 to measure temperature in °C, wind speed (m/s), and relative humidity (%).

### 2.4. Acoustic Analyses

#### 2.4.1. Fundamental Frequency (*f_o_*) Analysis

Data were segmented by defining the on- and off-set of each rumble using a customized annotation and labeling tool in S_Tools-STx 4.4.6 [60]. We used a custom-written STx script based on an autocorrelation method to automatically extract source-related *f_o_* parameters *f_o_ min*, *f_o_ max*, *f_o_ start*, *f_o_ end*, *f_o_ center*, and *f_o_ mean* (in Hertz). This is based on the total number of measuring points (Ntotal), determined by segment length and *f_o_ min* (*f_o_ min* determines the length of the analysis window that has to correspond to three *f_o_ min* periods; i.e., the lower *f_o_ min*, the longer the analysis window). We used a 75% overlap between successive kessel analysis windows with a bandwidth of 1 Hz. Only the number of frames with nonzero *f_o_* values (N*f_o_*) were considered for further analyses.

#### 2.4.2. Formant Frequency Location (F1, F2) Analysis and Vocal Tract Length

**Figure 3:**
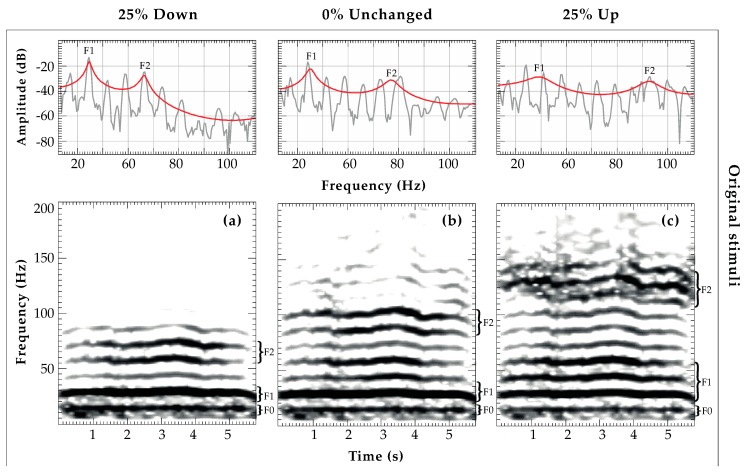

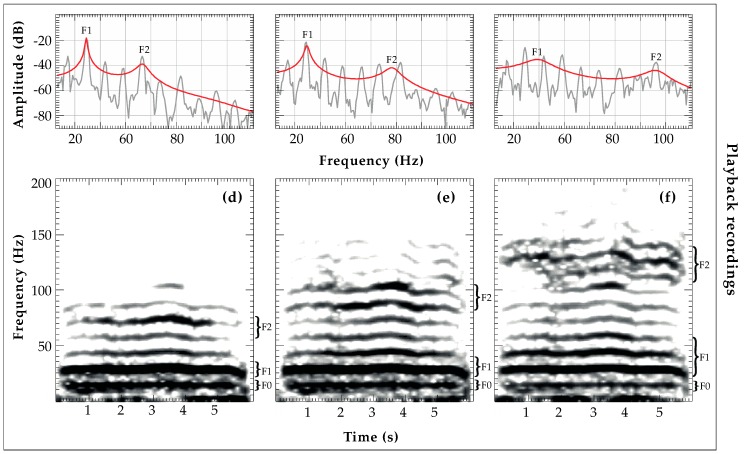
Narrow-band spectrograms and LPC spectra, indicating *f_o_*, *F1*, and *F2* location for each shift variant (25% Down, 0% Unchanged, and 25 % Up) of the original test data set (**a**–**c**) and the corresponding playback recordings from 50 m distance (**d**–**f**) (S_Tools-STx settings; analysis windows: Kaiser kessel, bandwidth: 2, overlap: 75%).

Prior to analysis all sound files were downsampled at 500 Hz, resulting in a frequency range of 0 to 250 Hz. Following the computation of LPC (Linear Predictive Coding)-smoothed spectrums in the range of 0 to 250 Hz using S_Tools-STx 4.4.6 (it was not possible to track formant frequencies continuously over the entire signal in Praat in our long-distance re-recordings), we measured the center frequency (in Hz) of the LPC spectral peaks, indicating formant positions. Differing experimental conditions in both habitats, such as higher background noise or wind speed, caused structural variation within the re-recorded sound signals. We therefore allowed a tolerance measurement of ±0.5 s for the re-recordings based on the original measuring point of the corresponding playback stimuli. That is, if the LPC peak of *F1* in the original stimulus was measured at 2.5 s, *F1* in the re-recorded stimulus could be measured between 2 s and 3 s. A comparison of LPC peak measurements of an original and re-recorded stimulus of all formant shift variants is illustrated in Figure 3.

In the present study, the second formant was less consistent than the first one, making it difficult to calculate FΔ and, therefore, a predicted VT length. Since VT length affects the overall formant frequency pattern, and the lowest formant potentially provides some information on VT length [45], we used *F1* to calculate ‘estimated VT lengths’ based on the equation $F_{1}=\frac{c}{4L}$, where *c* is the speed of sound (343.5 m/s [61]), and *L* is the length of the supralaryngeal vocal tract (assuming that the VT is a resonant tube open at one and closed at the other) [40].

#### 2.4.3. Amplitude Attenuation of Acoustic Features (SNR)

To assess the amplitude attenuation of *F1*, *F2*, and *f_o_* over distance in both habitats, we calculated the respective ‘Signal-to-noise Ratio’ (SNR) of these parameters with a custom-written script in S_Tools-STx. To examine environmental background noise levels alone, we extracted a 0.5 s segment directly before the onset and after the offset of each playback sequence. Root Mean Square (RMS) values for the environmental background noise were then computed at three frequencies (those corresponding to *F1*, *F2*, and *f_o_* in the playback segment) from an averaging of both noise segments. In parallel, for each playback segment, three RMS values were also measured, exactly at *F1*, *F2*, and *f_o_*. For each of these parameters, SNR was then determined by subtracting the RMS of the averaged noise segment (again only at the frequency of *F1* and not over the entire frequency range) from the RMS of the playback segment. This procedure was applied to determine SNR for *F1*, *F2*, and *f_o_* independently.

### 2.5. Statistical Analyses

To provide a way of determining ‘transmission success’ of playbacks through the environment, we calculated absolute numbers and percentages of successfully transmitted rumbles for each recording distance. Rumbles with a N*f_o_* detection rate below 60% were treated as insufficiently detected and hence discarded from further analysis. To assess the stability of the acoustic features, we compared *F1*, *F2*, *f_o_ mean*, and duration of rumbles with successful transmissions at 100 to 1500 m recording distance to rumbles obtained from the lowest distance, 50 m, by using nonparametric Mann–Whitney U tests (suited for non-normal distributed data).

Formants in nasal rumbles of savanna elephants encode information on maturity (i.e., age and body size) [37,38]. We therefore used VT length estimations (in m) to split our data set into two size groups (‘maturity group 1’ > 3 m and ‘maturity group 2’ ≤ 3 m). To examine differences between those groups per recording distance and per habitat, we performed Mann–Whitney U tests.

To assess attenuation of *F1*, *F2*, and *f_o_* with increasing distance from 50 to 1500 m, for each habitat, the SNR of each acoustic feature was regressed with recording distance using linear regression. The relationship between SNR and distance was then quantified by computing Cohen’s effect size index, f [62], for regression models using equation $f=\sqrt{\frac{R^{2}}{{1-R}^{2}}}$. All statistical tests were conducted using IBM SPSS statistics version 23 [63]. Significance levels were set at 0.05 and two-tailed statistics are reported.

## 3. Results

### 3.1. Transmission Success

**Figure 4:**
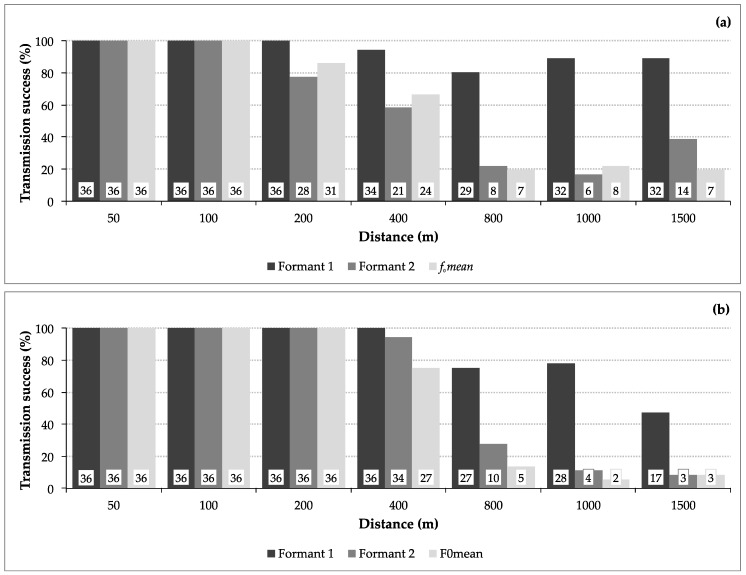
Transmission success (in percent) of *F1*, *F2*, and *f_o_ mean*: Comparison of each acoustic feature between the (**a**) dense and (**b**) open habitat over 50 to 1500 m distance. Values given at the bottom of the bars indicate the absolute number of recordings successfully transmitted per distance.

As expected, the propagation experiments conducted at the Addo Elephant National Park showed that the transmission success in two different habitats, densely vegetated and open, decreased with distance. The dense habitat resulted in a transmission success of 93.3% for *F1*, 59.1% for *F2*, and 59.1% for *f_o_ mean*, whereas in the open habitat *f_o_ mean* reached 57.5%, *F2* 63.1%, and *F1* 85.7%. The transmission of *F1* was most efficient compared to *F2* and *f_o_ mean* in both habitats (Figure 4).

**Figure 5:**
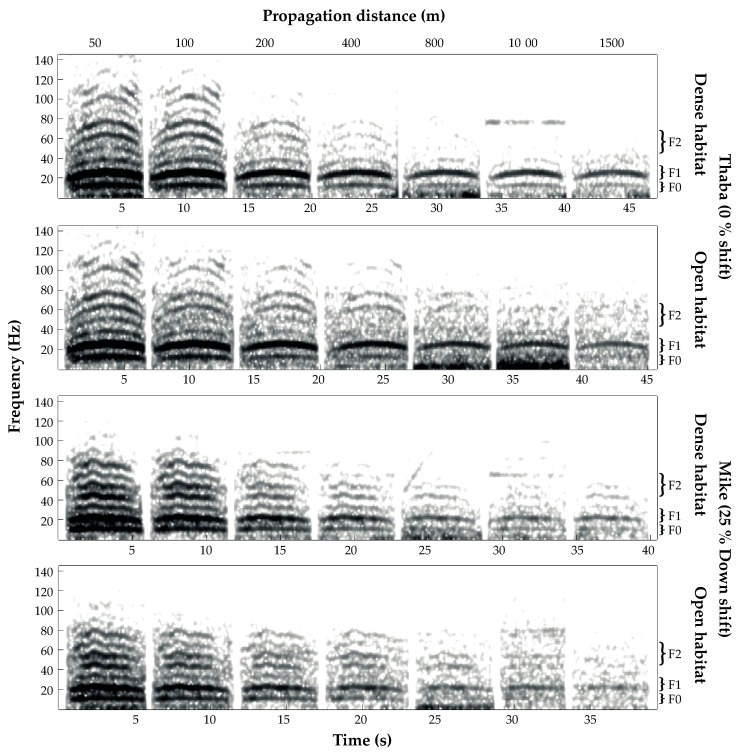
Spectrograms of concatenated 0% (from Thaba) and 25% downshifted (from Mike) re-recordings conducted from 50 to 1500 m for both dense and open habitat, respectively. Curly brackets indicate location for *F1* showing the highest transmission consistency, while *f_o_* and *F2* were less well-defined (S_Tools-STx settings; analysis windows: Kaiser kessel, bandwidth: 2, overlap: 75%).

Our comparisons of frequency parameters measured at 100 to 1500 m distance to those analyzed at 50 m revealed no significant differences (except for *F2* at 1000 m in the open habitat with *p* = 0.038, note the difference of N = 32 though). In particular, the signal length (duration) of the re-recordings revealed deviations, showing significant differences between 800 m, 1000 m, and 1500 m, respectively (Table 2). Visual examples for successful propagations of 0 % shifted and 25 % downshifted playback stimuli between both habitats can be identified from Figure 5.

### 3.2. Transmission of Size Information

VT length in savanna elephants is a reliable cue to body size [37,38]. By categorizing the recorded signals into two different size groups based on VT length estimations ranged from 2 to 5.47 m, our data showed significant differences for *F1* between rumbles simulating large (maturity group 1, MG 1) and small (maturity group 2, MG 2) male elephants. Dense habitat: *F1*_MG1_ = 23.55 ± 3.19 Hz (N = 140), *F1*_MG2_ = 35.28 ± 4.47 Hz (N = 95), *X^2^* = 169.073, df = 1, *p* < 0.001; Open habitat: *F1*_MG1_ = 23.67 ± 3.29 Hz (N = 125), *F1*_MG2_ = 35.40 ± 4.68 Hz (N = 91), *X^2^* = 157.263, df = 1, *p* < 0.001). 

Moreover, Kruskal–Wallis tests confirmed stable transmission of *F1*, with no significant differences of *F1* over distance for MG 1 at the dense (N = 140, *X^2^* = 2.569, df = 6, *p* = 0.861, r = 0.2) and open habitat (N = 125, *X^2^* = 4.855, df = 6, *p* = 0.563, r = 0.4). MG 2 was not statistically different in the dense habitat (N = 95, *X^2^* = 7.055, df = 6, *p* = 0.316, r = 0.7), while there was a significant change in the transmission of *F1* for MG 2 in the open habitat (N = 91, *X^2^* = 20.681, df = 6, *p* = 0.002, r = 2.167) over distance. However, performing pairwise comparisons using Dunn–Bonferroni post hoc corrections (adjusted significance level *p* = 0.002) did still not result in any significant differences for MG 2 in the open habitat (*p* > 0.002).

### 3.3. Amplitude Attenuation

**Figure 6:**
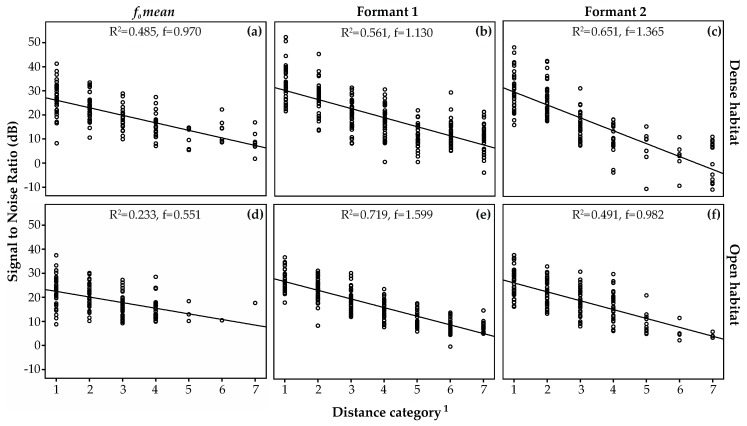
Signal-to-Noise ratio and regression lines for *f_o_ mean* (**a**,**d**), Formant 1 (**b**,**e**), and Formant 2 (**c**,**f**) per habitat over all recording distances (top three figures represent the densely vegetated and the three bottom figures the open habitat) show formant and *f_o_* attenuation with increasing distances. Cohen’s f indicates the effect sizes (f > 0.5 represents a strong effect size). ^1^ Distance category: 1 = 50 m, 2 = 100 m, 3 = 200 m, 4 = 400 m, 5 = 800 m, 6 = 1000 m, 7 = 1500 m.

Figure 6 shows that, overall, each acoustic parameter exhibited stronger attenuation with increasing distance for both habitats, with high effect sizes (f > 0.5) for all regression models. For instance, the SNR for *F1* at 1500 m was 10.4 ± 6.0 dB for the densely vegetated and 8.4 ± 2.2 dB for the open habitat. Mean SNR ± standard deviation for each recording distance (for *F1*, *F2*, and *f_o_*) and per habitat are listed in Table S3. Additional ANOVAs (temperature, wind speed, humidity, and height were not included as covariates due to inhomogeneity of regression coefficients; measurements are given in Table S4) testing for differences between both habitats revealed significant differences for *F1* only (N_Dense_ = 235, 19.1 ± 10.3 dB, N_Open_ = 216, 17.3 ± 8.0 dB; Anova: F = 4.339, df = 1, *p* = 0.038). In contrast, *F2* (N_Dense_ = 149, 18.6 ± 12.2 dB, N_Open_ = 159, 19.5 ± 7.6 dB; Anova: F = 0.576, df = 1, *p* = 0.448) and *f_o_ mean* (N_Dense_ = 148, 20.2 ± 7.8 dB, N_Open_ = 136, 19.9 ± 6.0 dB; Anova: F = 2.080, df = 1, *p* = 0.150) showed no differences.

## 4. Discussion

The field propagation experiments described in this paper reveal novel insights into the transmission of the infrasonic and long-ranging savanna elephant rumble. Our results show that the stability of spectral features in rumbles of four male savanna elephants, conveying measurable size-related information [37], prevailed over distances of up to 1.5 km under two different habitats. However, it remains to be investigated whether savanna elephants can indeed perceive rumbles with the obtained SNR values. Sounds are processed within listener’s auditory system, but in savanna elephants, to our knowledge there is no data available on their hearing threshold or other hearing capabilities, such as directional hearing, sound localization, antimasking mechanisms, critical bandwidths, and critical ratios [64]. In addition, hearing sensitivity might as well be influenced by sex and age [65,66,67,68,69] in this species.

Previous research using playback experiments demonstrated that savanna elephant rumbles encode acoustic information about sex, reproductive state, and even social identity [38,70,71,72,73]. Savanna elephants are capable of not only recognizing rumbles of other family and bond group members within their population but also of discriminating calls from conspecifics they encountered more or less frequently [71]. It has also been shown that savanna elephants detect contact rumbles broadcasted at distances ranging between 0.5 and 2.5 km, revealing their ability to recognize rumbles and to assign them to family members at distances up to 1.5 km [33,54]. Nonetheless, as vocalizations propagate through an animal’s natural environment, they degrade over distance in various acoustic parameters such as amplitude (source intensity) and frequency patterns. This can potentially affect the detection of acoustic features and eventually hinder savanna elephants’ discriminative abilities. McComb et al. [54] found that *F2* (harmonics region around 115 Hz) was the most prominent and persistent acoustic feature measured in rumbles over distance. They suggested accordingly that frequencies above the infrasonic range play an important role for social recognition in savanna elephants. Our findings provide a key difference to these previous results, as our transmission profiles showed that efficiency and persistency were highest for *F1* and a clear loss of higher harmonics in the *F2* region, as shown in forest elephants [74]. Yet, importantly, our results support McComb et al.’s conclusion highlighting the relevant role of frequencies above infrasound in savanna elephant communication (given that *F1* is usually around 25 to 35 Hz). We observed a similar degradation pattern between *F2* and *f_o_*, where contour detection in both experimental habitats was most consistent between 50 and 400 m, but dropped increasingly from 800 m to 1.5 km.

In contexts of male competition, formant frequencies have been described in several mammal species (e.g., red deer [42] and koala [75]) as robust acoustic indicators of a caller’s body size (more so than *f_o_* [45]). In a previous research project we revealed that formant frequencies generated via the nasal vocal tract serve as an honest cue to the maturity status (age and body size) in male savanna elephants [37]. We show that this information is likely to be transmitted over distance since significant differences between both bull size categories (≤3 m and >3 m) remained in both habitats (yet, our sample size is considerably small). Note, however, that VT length estimations, and hence body size predictions in the present study, were based on individual *F1* frequencies instead of formant spacing because *F2* transmission was inconsistent and difficult to identify, particularly at larger distances. Earlier studies raised concerns about the reliability of using one formant as a single cue for VT length, due to environmental factors potentially degrading the chosen frequency band and to sensitivity to possible deviations from the uniform tube assumption [45,76]. Therefore, this might lead to imprecise VT length estimations. Formant dispersion, in contrast, relies on redundant formant spacing patterns and is considered to be more resistant to adverse environmental distortion factors and individual formant variability [45]. Consequently, most playback studies examining the relevance of formants used formant dispersion as a measure of size discrimination [43,44,77,78]. Other perceptual studies, however, showed that some non-human primates place more weight on the position of *F1* than *F2* [79]. Since *F2* was measured inconsistently at longer recording distances, we estimated VT length using *F1* locations. Furthermore, we provide evidence that, particularly, *F1* frequencies propagated with higher consistency than *f_o_* and *F2* across and between all measuring points in both tested habitats. Our results demonstrate that *F1* in nasally emitted social rumbles may travel up to 1.5 km and underline their informative value and potential relevance for long-distance communication where body-size assessment matters.

The spectral and temporal acoustic characteristics of a sound determine how far it will travel through the environment [9,11]. However, sound propagation and sound detectability are also critically determined by the habitat structure, ambient noise source, and local atmospheric conditions (such as temperature, wind, and humidity). These can either favor or impede sound propagation [7]. The speed of sound in any given terrestrial environment depends on air temperature. For instance, the approximate speed of airborne sound at 20 °C is 343.5 m/s (using the formula c=331.4+0.607×ambient temperature (°C)[61]). Rising temperatures boost sound velocity. Humidity may also increase the speed of sound, e.g., at 100% relative humidity and 20 °C, sound velocity is approximately 0.3% greater than at 30% relative humidity [12,61]. Furthermore, empirical data and computer modeling of the African savannah revealed that optimal conditions for elephant low-frequency sound propagation are given under low wind and cool temperatures, particularly 1–2 h after sunset when air at ground level cools down rapidly [80]. Therefore, Garstang et al. [80] proposed that savanna elephants adapt their long-ranging and low-frequency calling rates to atmospheric conditions. These authors suggested that, by making use of near-surface temperature inversions, savanna elephants can increase their call propagation ranges considerably (up to 10 km under ideal atmospheric conditions) [80]. However, whether they actually do adjust their vocalizations to optimal atmospheric conditions is not supported by any data provided so far.

To date, the behavioral responses of wild savanna elephants to playback stimuli have been experimentally documented at a maximum distance of 2.5 km only [54] (but were estimated to be audible to conspecifics at least 4 km away from the source using data extrapolations [33]). Furthermore, wind can be directly related to turbulence and cause more than mere attenuation along a sound’s broadcast direction [7]. Our experiments were thus always conducted under low wind conditions. Nonetheless, although wind speed measurements were conducted at the site of the re-recordings, we cannot rule out that sudden wind gusts between both vehicles might have affected the broadcasted signals.

Differences in vegetation, topography, and atmospheric conditions can influence sound transmission via reverberation, amplitude fluctuations, and attenuation at all frequencies, and result in temporal and spectral degradation of various degree [7,8,9,10,11,12]. While we did not find any frequency-dependent differences in propagation between open and dense habitats, we did observe deviations in call duration at larger recording distances. However, to what extent temporal degradation processes might play a role for signal detection or distance assessment requires further investigation.

Signal detection and recognition strongly depend on the signal-to-noise ratio (SNR), a measure of a signal’s maximum amplitude relative to ambient noise. Therefore, ambient noise level (in addition to atmospheric distortion factors) also plays an important role in sound detection [9]. However, it is suggested that signal amplitude alone is not entirely reliable cue for distance assessment and that temporal and/or frequency-specific structures are likely to serve as more complex acoustic cues [17,18,19]. Our analyses of rumble broadcast data confirmed the prediction for degradation trends for *F1*, *F2*, and *f_o_ mean*: transmission efficiency and intensity of rumbles decrease progressively with increasing distance. Not only unnoticed wind gusts between both vehicles during the experiments but height differences might also have played a role in sound transmission through changes in SNRs. The terrain of the open habitat showed greater height differences between the subwoofer and the respective recording distance than the more densely vegetated habitat. However, if this effect on SNR was present it appears negligible as, overall, we found no noticeable differences in transmission efficiency between both habitats. However, low-frequency sound waves tend to be enhanced particularly when traveling above porous surfaces (such as soil or sand) because larger sound wavelengths penetrate the ground pores less [16]. Although this insight was not obtained experimentally, we cautiously assume that constructive ground effects in the tested habitat could likely enforce long-distance rumbles by superimposed reflections, as it has been recently suggested for the densely vegetated habitat of forest elephants [74].

The pioneering field playback studies in the early 1990s and 2000s conducted by Langbauer et al. [33] and McComb et al. [54] provided first evidence on the nature of rumbles and their importance for long-distance communication. Our results are in general in agreement with savanna elephant’s large body size allowing the production of low-frequency and high-intensity rumbles, both favoring propagation to distances over which this species communicates. McComb et al. [54] found that frequency peaks in rumbles re-recorded over several hundred meters remained most prominent and stable in the *F2* region. Our results indicate that formants likely serve as long-range signal in savanna elephants, instead of being merely relevant for short- to medium-range communication, as previously suggested [54]. Importantly (both from a theoretical and practical standpoint), most of the acoustic energy in our study was concentrated and persisted at the *F1* frequency position, compared to *F2* and *f_o_ mean*. The use of female rumbles by McComb et al. [54] and male rumbles in our study is a potential factor explaining deviations between both studies, i.e., sexual vocal dimorphism in social rumbles [38], which however requires further testing. Finally, differences between our findings might merely reflect the use of different equipment (e.g., speaker, variation of frequency responses between microphones and analog DAT recorders [54]).

## 5. Conclusions

In conclusion, our study describes how the environment can influence propagation of rumbles and what kind of information might be transmitted successfully over distance. Future field playback experiments using formant-shifts, and thus different size variants of male rumbles, could provide information on two aspects: assessing the size discrimination abilities of savanna elephant bulls based solely on vocalizations, and identifying how rumble degradation over distance relates to its perception in this species.

## Figures and Tables

**Table 1:** Study sites and age for each study subject.

Location, Year of Data Collection	Individual	Age (Years)	Approx. Shoulder Height (m)
Pilanesberg, 2014	Mike	∼29	3.20 m
Bela Bela, 2014	Chishuru	∼18	2.40 m
Hazyview, 2014	Medwa	∼19	2.60 m
Addo Elephant Back Safaries, 2016	Thaba	∼31	3.25 m ^1^

∼ indicates that the exact birth date is unknown. ^1^ Shoulder height measured in 2014.

**Table 2:** Acoustic features recorded and measured at 50 m compared to all other distances ranging from 100–1500 m using Mann–Whitney U tests.

Habitat	Distance (m)	Acoustic Features																		
		Formant 1 (Hz)					Formant 2 (Hz)					*f_0_ mean* (Hz)					Duration (s)			
		N	TS	U	Z	P	N	TS	U	Z	P	N	TS	U	Z	P	N	U	Z	P
**Dense**	**50**	36	100	-	-	-	36	100	-	-	-	36	100	-	-	-	36	-	-	-
	**50–100**	36	100	636	−0.135	0.892	36	100	646	−0.028	0.978	36	100	645	−0.039	0.969	36	622	−0.298	0.765
	**50–200**	36	100	629	−0.220	0.826	28	78	454	−0.683	0.494	31	86	542	−0.201	0.840	36	604	−0.501	0.616
	**50–400**	34	94	587	−0.300	0.764	21	58	356	−0.364	0.716	24	67	364	−1.027	0.305	36	557	−1.031	0.303
	**50–800**	29	81	501	−0.277	0.782	8	22	120	−0.730	0.465	7	19	72	−1.778	0.075	32	376	−2.464	0.014
	**50–1000**	32	89	556	−0.252	0.801	6	17	72	−1.294	0.196	8	22	129	−0.442	0.659	36	442	−2.320	0.020
	**50–1500**	32	89	561	−0.184	0.854	14	39	200	−1.124	0.261	7	19	117	−0.313	0.754	36	432	−2.433	0.015
**Open**	**50**	36	100	-	-	-	36	100	-	-	-	36	100	-	-	-	36	-	-	-
	**50–100**	36	100	642	−0.068	0.946	36	100	637	−0.130	0.897	36	100	623	−0.282	0.778	36	621	−0.304	0.761
	**50–200**	36	100	608	−0.451	0.652	36	100	646	−0.023	0.982	36	100	645	−0.039	0.969	36	644	−0.051	0.960
	**50–400**	36	100	610	−0.434	0.665	34	94	588	−0.282	0.778	27	75	411	−1.042	0.297	36	613	−0.400	0.689
	**50–800**	27	75	450	−0.500	0.617	10	28	118	−1.665	0.096	5	14	62	−1.117	0.264	34	435	−2.086	0.037
	**50–1000**	28	78	453	−0.697	0.486	4	11	26	−2.074	0.038	2	6	36	0.000	1.000	31	350	−2.616	0.009
	**50–1500**	17	47	298	−0.162	0.871	3	8	30	−1.265	0.206	3	8.	45	−0.475	0.635	21	138	−3.979	0.000

N = number of analyzed rumbles at 50–1500 m; TS = Transmission success of Formant 1, Formant 2 and *f_o_ mean* in percent (%); U = Mann–Whitney U test U-score; Z = Mann–Whitney U test Z-score, *p* = significance level (*p* = 0.05).

## Supplementary Materials

The following are available online at http://www.mdpi.com/2076-2615/8/10/167/s1; Table S1: Formant-related features measured in S_Tools-STx; Table S2: GPS coordinates and topographic height per habitat and recording distance; Table S3: Signal-to-noise ratios: mean, ± standard deviation (Stdev); Table S4: Atmospheric conditions (mean ± standard deviation) for each recording distance and habitat.
