# Supplementary material for: Field Propagation Experiments of Male African Savanna Elephant Rumbles: A Focus on the Transmission of Formant Frequencies
Source: Animals (Basel). 2018 Sep 30;8(10):167. doi: 10.3390/ani8100167 (PMC6210294; doi:10.3390/ani8100167)
Supplement: Supplementary file 1 [file animals-08-00167-s001.pdf]

Article

Field Propagation Experiments of Male African Savanna Elephant Rumbles: A Focus On the Transmission of Formant Frequencies – Supplementary tables

Table S1. Formant-related features measured in S_Tools-STx.

Individual	Formant-shift	Duration (s)	Formant 1 (Hz)	Formant 2 (Hz)	Formant Dispersion (Hz)
Chishuru	25% Down	5.748	25.29	73.10	47.8
	0% Unchanged	5.748	33.78	97.72	63.9
	25% Up	5.748	42.20	128.48	86.3
Medwa	25% Down	4.222	23.59	73.95	50.4
	0% Unchanged	4.222	31.10	98.37	67.3
	25% Up	4.222	39.98	120.05	80.1
Mike	25% Down	5.034	15.98	48.57	32.6
	0% Unchanged	5.034	21.18	67.33	46.2
	25% Up	5.034	26.50	86.73	60.2
Thaba	25% Down	5.846	20.67	50.53	29.9
	0% Unchanged	5.846	26.96	66.82	39.9
	25% Up	5.846	32.51	90.10	57.6

Table S2. GPS coordinates and topographic height per habitat and recording distance. Height difference provides information on differences in meter between the respective recording distance and the subwoofer's position.

Habitat	Recording distance (m)	Latitude, Longitude	Ellipsoidal height (Altitude)	Topographic height (m)*	Height difference (m)
Dense	0	33°27'52.4"S 25°44'10.8"E	194	184.8	-
	0†	33°27'53.8"S 25°44'10.0"E	189	179.8	-
	0§	33°27'54.8"S 25°44'09.7"E	195	185.8	-
	50	33°27'50.8"S 25°44'11.2"E	193	183.8	1
	100	33°27'49.2"S 25°44'12.0"E	193	183.8	1
	200	33°27'46.1"S 25°44'13.4"E	191	181.8	3
	400	33°27'40.0"S 25°44'15.6"E	197	187.8	3
	800	33°27'27.0"S 25°44'13.8"E	181	171.8	13
	1000	33°27'21.5"S 25°44'10.0"E	189	179.8	0
	1500	33°27'07.3"S 25°44'00.8"E	195	185.8	0
Open	0	33°29'34.8"S 25°49'38.5"E	346	336.8	-
	50	33°29'35.0"S 25°49'36.5"E	346	336.8	0
	100	33°29'34.3"S 25°49'34.5"E	341	331.8	5
	200	33°29'31.2"S 25°49'32.1"E	335	325.8	11
	400	33°29'24.8"S 25°49'29.0"E	328	318.8	18
	800	33°29'38.9"S 25°49'07.6"E	310	300.8	36
	1000	33°29'42.5"S 25°49'00.7"E	307	297.8	39
	1500	33°29'53.8"S 25°48'44.5"E	287	277.8	59

†corresponds to the 0 m position of the subwoofer for 1000 m

§corresponds to the 0 m position of the subwoofer for 1500 m

*calculation for topographic height were performed using the Geoid height calculator provided by UNAVCO, Inc. [45]

Table S3. Signal-to-Noise Ratios: Mean, ± standard deviation (Stdev). N represents the number of analyzed acoustic features for each recording distance at the densely vegetated and open habitat.

Distance (m)	Signal-to-Noise Ratio (dB)																	
	Dense habitat									Open habitat								
	Formant 1			Formant 2			*f*₀ *mean*			Formant 1			Formant 2			*f*₀ *mean*		
	Mean	Stdev	N	Mean	Stdev	N	Mean	Stdev	N	Mean	Stdev	N	Mean	Stdev	N	Mean	Stdev	N
50	32.7	7.7	36	30.3	7.7	36	27.6	6.4	36	27.2	4.5	36	26.1	5.7	36	22.8	6.3	35
100	26.9	6.6	36	26.3	6.7	36	23.2	5.3	36	23.7	4.9	36	22.3	5.0	36	20.6	5.1	35
200	20.6	6.2	36	15.2	5.6	28	18.6	4.3	29	19.5	4.8	36	18.4	5.9	36	17.2	4.7	35
400	17.1	6.8	34	10.4	6.0	21	15.2	5.7	24	15.1	4.0	36	16.4	5.7	34	15.0	4.5	26
800	11.0	5.0	29	6.7	7.9	8	10.8	4.0	7	10.7	3.3	27	9.8	4.8	10	13.8	4.2	3
1000	11.9	7.1	32	2.5	6.6	6	13.2	4.6	9	8.3	3.0	28	6.1	4.0	4	10.5	.	1
1500	10.4	6.0	32	1.8	7.9	14	8.8	4.7	7	8.4	2.2	17	4.6	1.3	3	17.7	.	1

Table S4. Atmospheric conditions (mean ± standard deviation) for each recording distance and habitat.

Distance	Humidity (%)		Temperature (°C)		Wind speed (m/s)	
	Dense	Open	Dense	Open	Dense	Open
50	70.4 ± 12.2	51.3 ± 20.5	20.9 ± 4.2	26 ± 3.8	0	0.5 ± 0.5
100	64 ± 6.6	53.3 ± 20.7	23 ± 2	24.5 ± 3.1	0.2 ± 0.2	0.2 ± 0.4
200	55 ± 10.2	35.8 ± 13.6	24.5 ± 2.5	24.2 ± 2.6	0	0 ± 0.1
400	50.7 ± 7.1	52.8 ± 13.8	26.7 ± 1.5	24.9 ± 0.9	0.2 ± 0.3	0 ± 0.1
800	49.1 ± 5	51.6 ± 14.6	27.9 ± 1.5	24.6 ± 1.5	0.4 ± 0.5	0.2 ± 0.4
1000	48.3 ± 4.3	59.5 ± 16.2	27.7 ± 1.8	21.4 ± 2.3	0.2 ± 0.4	0.2 ± 0.4
1500	45.4 ± 2.5	54.1 ± 17.6	28.5 ± 0.6	21.1 ± 2.5	0 ± 0.1	0
